# Fertility Preservation and Ovarian Hyperstimulation Syndrome Management in Cancer Care: A Pathophysiological Perspective on Gonadotropin-Releasing Hormone Agonists and Antagonists

**DOI:** 10.3390/pathophysiology31020021

**Published:** 2024-06-07

**Authors:** Giuliano Bedoschi, Caroline Ingold, Paula Andrea Navarro

**Affiliations:** 1 Department of Gynecology and Obstetrics, Reproductive Medicine Division, Ribeirão Preto Medical School, University of São Paulo, Ribeirão Preto 14048-900, Brazil; drapaulanavarro@gmail.com; 2 Department of Collective Health, Sexual, Reproductive Health and Population Genetics, Faculdade de Medicina do ABC, Santo André 09060-870, Brazil; caroline.c.ingold@gmail.com

**Keywords:** fertility preservation, ovarian hyperstimulation syndrome, gonadotropin-releasing hormone, quality of life, neoplasms, cryopreservation, drug therapy

## Abstract

This narrative review delves into the evolving landscape of fertility preservation techniques, with a particular focus on their use in patients undergoing oncology treatment that carries a risk of ovarian insufficiency. Advances in established methods such as cryopreservation of oocytes and embryos are highlighted, and the increasing use of gonadotropin-releasing hormone (GnRH) agonists is discussed. The review also addresses the complexities and controversies associated with these approaches, such as the ‘flare-up’ effect associated with GnRH agonists and the potential of GnRH antagonists to reduce the risk of ovarian hyperstimulation syndrome. Despite advances in fertility preservation, the report highlights the challenges we face, including the need for personalized treatment protocols and the management of associated risks. It calls for continued research and collaboration between healthcare professionals to refine these techniques and ultimately improve reproductive outcomes for patients facing the prospect of fertility-impairing treatment.

## 1. Introduction

Controlled ovarian stimulation (COS) is an essential component of assisted reproductive technologies (ART), which primarily aim to improve oocyte yield during retrieval. Although this strategy increases reproductive success, it also carries the risk of ovarian hyperstimulation syndrome (OHSS). While OHSS can occur spontaneously [1], it is primarily an iatrogenic consequence of COS, particularly in women undergoing ART. Moderate-to-severe OHSS occurs in approximately 3 to 10% of ART cycles, although this figure can rise to 20% in high-risk groups [2].

Effective OHSS risk management depends on recognizing the key predictors [3]. Elevated anti-Mullerian hormone levels and an increased number of antral follicles are important risk indicators. In addition, a high ovarian response, characterized by numerous dominant follicles, elevated estradiol levels at the time of ovulation induction, and the retrieval of several oocytes, serves as a reliable marker of OHSS risk. Personalizing ovarian stimulation protocols based on these risk factors is critical to protecting patient health.

Various fertility preservation methods are currently available to increase the likelihood of future pregnancy in patients receiving cancer treatment who are at risk of ovarian failure after completion of therapy [4]. Some established methods of fertility preservation, including oocyte and/or embryo cryopreservation, require COS as a crucial part of the process. For these patients, the problem of OHSS becomes even more complex. In addition to the physical and emotional stress of the cancer diagnosis and impending treatment, these patients face the additional risks of OHSS, including an increased risk of thromboembolism, possible delays in cancer treatment, and increased emotional stress.

Recently, the simultaneous use of several fertility preservation strategies in individual patients has been increasingly used to optimize the prospects of future pregnancies. In this context, the administration of long-term gonadotropin-releasing hormone (GnRH) agonists is frequently observed in women who have undergone oocyte retrieval for oocyte and/or embryo cryopreservation. However, there are reports of OHSS in women who have undergone COS followed by treatment with long-term GnRH agonists [5,6], despite having no other identifiable risk factors for this complication. A comprehensive understanding of both the pathophysiology of OHSS and the various fertility preservation techniques is crucial, especially for oncology patients. This knowledge enables healthcare professionals to develop personalized, patient-tailored plans that effectively minimize both the incidence and severity of OHSS.

This narrative review addresses the pathophysiology of OHSS, especially in the context of the use of GnRH agonists in cancer patients. Our discussion pivots on discussing innovative strategies, particularly emphasizing the potential of GnRH antagonists as a safer and more effective alternative for managing OHSS risk in this particular patient population. By exploring these avenues, we aim to align fertility preservation efforts with the broader health and treatment goals of cancer patients and ensure a balanced and safe approach to their reproductive journey.

## 2. Search Strategy

Our narrative review was conducted through a systematic search of electronic databases such as PubMed, Scopus, and Web of Science to find the relevant literature published up to December 2023. The search strategy was designed to include a broad range of terms and keywords related to “gonadotropin-releasing hormone (GnRH) agonists and antagonists”, “fertility preservation”, “ovarian hyperstimulation syndrome” and “cancer treatment”. The reference lists of identified articles were also checked to capture additional studies not found in the database searches.

We used a combination of terms from the Medical Subject Headings (MeSH) and free text terms with appropriate Boolean operators to ensure a comprehensive search of the literature. The search was limited to articles published in English. While our search was not exhaustive, as in a systematic review, it aimed to provide a thorough representation of the current landscape of the topic. The selected articles were then critically reviewed and synthesized to reflect the continuum of evidence and opinion in the field, from basic research to clinical practice guidelines.

### 2.1. Impact of Oncologic Treatment

Cancer treatments, including radiotherapy and chemotherapy, have significantly improved survival rates and quality of life for patients. This section delves into the specific impacts of radiotherapy and chemotherapy on reproductive health, highlighting the risks and mechanisms of harm.

### 2.2. Radiotherapy

The risk of reproductive harm from radiotherapy increases with higher radiation doses. A dose of 5 to 20 grays (Gy) to the ovaries can severely impair gonadal function. A dose of 2 Gy can damage 50% of the ovarian reserve [7]. Damage also occurs from scatter radiation, even if the ovaries are not directly in the radiation field. Pelvic/abdominal radiotherapy can harm uterine function, leading to infertility, miscarriage, and preterm birth.

### 2.3. Chemotherapy

Chemotherapy adversely affects ovaries, especially with alkylating agents like cyclophosphamide, increasing the risk of gonadal dysfunction. Agents like doxorubicin, cisplatin, and carboplatin also pose significant risks. The main mechanism is the accelerated loss of germ cells due to direct toxic effects on oocytes and granulosa cells [8]. This risk is higher in older women due to a reduced pool of primordial follicles.

### 2.4. Fertility Preservation

The prospect of infertility due to oncologic treatments poses a significant concern for cancer patients, necessitating the implementation of fertility preservation strategies. These strategies aim to protect reproductive potential without compromising the effectiveness of cancer therapies. 

### 2.5. Counseling

Proper counseling about infertility risks and effective communication with oncologists are vital for timely fertility preservation without delaying cancer treatment. In the U.S., many women with cancer express interest in fertility, but only 68% receive adequate information before treatment. An online survey found that 51% of young breast cancer survivors were concerned about future infertility, and 18% reported that these concerns influenced their treatment decisions [9].

### 2.6. Preventive Measures to Reduce Reproductive Damage

Several measures can minimize reproductive damage:

- Reducing radiation dose or field: Modern equipment allows for precise targeting of tumor cells.
- Ovarian transposition: Surgically repositioning ovaries out of the radiation field.
- Ovarian shielding: Using external protective shields during radiation.
- Using less gonadotoxic chemotherapy agents.
- Gonadal suppression with GnRH agonists to protect dividing cells during chemotherapy, though its efficacy remains debated.
- Oocyte and embryo cryopreservation
- Ovarian tissue cryopreservation

### 2.7. Oocyte and Embryo Cryopreservation

Oocyte and embryo cryopreservation are established fertility preservation techniques. Both require controlled ovarian stimulation (COS) and oocyte retrieval, taking 2–4 weeks. Embryo cryopreservation is a long-standing practice with well-documented outcomes comparable to fresh embryos. It requires a partner or donor sperm, involving ethical and legal considerations. Oocyte cryopreservation is newer but advantageous as it does not require a partner. Current survival rates are 90%, with similar fertilization and pregnancy outcomes to fresh oocytes.

### 2.8. Ovarian Tissue Cryopreservation

Ovarian tissue cryopreservation is an experimental technique involving laparoscopic biopsies or oophorectomy. Ideally performed before chemotherapy/radiotherapy, though post-exposure cryopreservation is possible for young women with viable follicles. Slow freezing is preferred over vitrification. Over 200 live births have been reported. It is suitable for prepubertal girls or women who cannot undergo ovarian stimulation.

Successful transplantation can restore hormonal function and fertility, whether performed orthotopically (in the pelvis) or heterotopically (e.g., in the arm or abdomen). The risk of reintroducing malignant cells is a concern, contraindicating this method in leukemia but considered safe for Hodgkin’s lymphoma. Each case requires an individual risk assessment with oncologists. Malignant cell screening is recommended before transplantation.

## 3. Pathophysiology of Ovarian Hyperstimulation Syndrome

Controlled ovarian stimulation (COS) protocols are a cornerstone of assisted reproductive technologies that aim to induce the development of multiple follicles in the ovaries. This process begins with the administration of gonadotropins, hormones that stimulate the ovaries to grow multiple follicles. To prevent a premature increase in luteinizing hormone (LH), which could lead to premature ovulation and reduce the effectiveness of fertility treatment, progestins, GnRH agonists, or antagonists are used. These agents modulate the body’s own hormone secretion and ensure that the time of ovulation can be precisely controlled. Finally, human chorionic gonadotropin (hCG) or a GnRH agonist is administered to trigger the final maturation of the follicles and prepare them for oocyte retrieval.

Ovarian hyperstimulation syndrome is a complex and multifaceted condition that occurs primarily as a complication of COS. The pathophysiology of OHSS is multifaceted and involves a number of biological processes ranging from vascular changes to cytokine-mediated responses.

Central to the pathophysiology of OHSS is the phenomenon of marked arteriolar vasodilation associated with increased capillary permeability. This vascular response leads to a significant shift of fluid from the intravascular to the extravascular space and sets the stage for the clinical manifestations of the syndrome. A key factor in the development of OHSS is the administration of hCG, which is commonly used to final follicular maturation. Human chorionic gonadotropin has a direct effect on the production of vascular endothelial growth factor (VEGF), a critical component in angiogenesis and increasing vascular permeability [10]. The severity of OHSS has been directly linked to VEGF levels, emphasizing the fundamental role of this growth factor in the pathogenesis of the syndrome [11].

Controlled ovarian stimulation is accompanied by a biochemical cascade characterized by the overproduction of pro-inflammatory and vasoactive cytokines [12]. These cytokines primarily include interleukin-1β (IL-1β), IL-6, IL-8, and tumor necrosis factor α (TNF-α), which play a crucial role in enhancing vascular permeability and underscore the inflammatory nature of OHSS.

The resulting increased vascular permeability and the consequent extravasation of protein-rich fluid into the extravascular space are responsible for the characteristic symptoms of OHSS, such as bloating, ascites, and generalized edema. This fluid shift not only underlies these clinical features but also carries the risk of more serious complications, including hypovolemic shock and renal dysfunction.

## 4. Clinical Presentation of Ovarian Hyperstimulation Syndrome

Ovarian hyperstimulation syndrome manifests as a constellation of symptoms primarily caused by marked vascular permeability and ovarian enlargement following COS.

Early signs of OHSS typically include a distended abdomen and mild discomfort due to ovarian enlargement with cyst formation, which can reach a diameter of 12–25 cm [13]. These enlarged cystic ovaries carry the risk of rupture or hemorrhage leading to peritonitis and are prone to complications such as ovarian torsion [14].

Increased capillary permeability leads to a significant shift of fluid into the third space, resulting in intravascular volume depletion. This underlies various clinical features that increase in severity with increasing involvement of multiple organ systems. Ascites, characterized by the accumulation of fluid in the abdominal cavity, often serves as the first indicator of OHSS and leads to abdominal pain and increased intra-abdominal pressure (IAP). Elevated IAP can develop into intra-abdominal hypertension (IAH) and, in severe cases, abdominal compartment syndrome, which is characterized by a persistent elevated IAP and is associated with organ dysfunction [10].

Oliguria is often an early sign of IAH. Impaired intra-abdominal venous outflow contributes to renal, intestinal, and hepatic edema, which subsequently leads to liver damage, paralytic ileus, and severe gastrointestinal symptoms. Elevated liver enzyme levels, such as aspartate aminotransferase and alanine aminotransferase, are common in severe cases. Acute renal failure in OHSS is a complex, multifactorial condition often characterized by a hypo-osmolar state that manifests as hyponatremia due to low serum osmolality. Contributing factors include intravascular volume depletion, renal edema due to increased capillary permeability, abdominal compartment syndrome due to increased intra-abdominal pressure, and obstructive uropathy caused by enlarged ovaries [15].

Patients with OHSS may present with leukocytosis, increased hematocrit, and thrombocytosis, indicating hemoconcentration and inflammation. Such hemoconcentration predisposes patients to hypercoagulability, with thrombotic events occurring in up to 10% of severe cases [16]. The risk is further increased by factors such as compression of the pelvic vessels and underlying thrombophilia. Thrombosis primarily affects the venous system, although arterial embolism is also a problem [17].

Patients diagnosed with ovarian hyperstimulation syndrome often present immunodeficiency with reduced levels of the immunoglobulins IgA and IgG, which increases the risk of infection. A significant proportion of hospitalized patients may develop febrile episodes, with urinary tract infections, pneumonia, and other site-specific infections being common [18].

In critical cases, patients may experience hypovolemic shock, septic shock, distributive shock due to severe inflammation, or cardiogenic shock due to complications such as pericardial effusion or pulmonary embolism [15]. Pericardial effusions, which only occur in a minority of cases, rarely lead to tamponade [19].

## 5. Interventions to Reduce the Risk of OHSS during COS

A number of interventions have been developed to reduce the risk of OHSS during COS, targeting specific elements of the pathophysiology.

Firstly, mild stimulation protocols are important. These involve the administration of daily FSH doses of 150 IU or less, a less aggressive approach compared to conventional methods. The advantage of these milder protocols is that they are associated with fewer adverse events, particularly a lower risk of OHSS. This is mainly due to the fact that the likelihood of OHSS is directly related to the intensity of ovarian stimulation [20].

Another important intervention is the use of GnRH antagonists. These agents competitively bind to the natural GnRH receptors and immediately and reversibly suppress the release of gonadotropin. This prevents a premature LH surge during COS, allowing for shorter treatment protocols and lower gonadotropin doses. This not only reduces costs but also significantly reduces the risk of OHSS by limiting gonadotropin overdose [21].

The role of hCG in final follicular maturation is also crucial, as it is closely linked to OHSS. Theoretically, bypassing hCG can mitigate OHSS-related complications, as OHSS cases without hCG are rare [22]. Options include either canceling the cycle prior to hCG administration or substituting GnRH agonists for hCG, thereby reducing the hCG dose [23]. However, these approaches can be emotionally and financially stressful [3].

In addition, the ‘freeze-all’ strategy has proven to be a viable option. In this approach, all retrieved oocytes/embryos are cryopreserved after retrieval, and embryo transfer is postponed to a later, non-stimulated cycle. By limiting exposure to hCG and avoiding prolonged exposure to natural hCG during pregnancy, which can exacerbate OHSS, this strategy serves as a preventative measure [24].

Finally, cabergoline, a dopamine agonist, has demonstrated its potential to reduce vascular hyperpermeability, a key aspect of OHSS pathophysiology. In animal studies, cabergoline demonstrated its ability to inhibit the VEGF-2 receptor, thereby reducing vascular hyperpermeability without impairing luteal angiogenesis. These findings have led to clinical studies investigating the efficacy of cabergoline in the prevention of OHSS [25].

## 6. OHSS in Cancer Patients Referred for Fertility Preservation

Women of reproductive age who have been diagnosed with cancer face a double challenge: fighting their disease and preserving their fertility. The risk of ovarian failure and infertility resulting from the adverse effects of oncologic treatments such as chemotherapy and radiation underscores the importance of fertility preservation as an integral aspect of patient care [8]. For this reason, leading scientific societies have formulated comprehensive guidelines to address this complicated aspect of oncologic treatment [26,27,28,29].

Early referral to a reproductive medicine specialist is a common thread in these guidelines, emphasizing the need for timely fertility preservation without delaying oncologic treatment. Established methods such as cryopreservation of oocytes and embryos are crucial in preserving the fertility of cancer patients, with strategies such as random start protocols and the use of aromatase inhibitors in hormone-sensitive cancers tailored to the needs of patients. The innovative cryopreservation of ovarian tissue has led to promising results. More than 200 live births have been reported, highlighting the significant advances in autotransplantation and fertility restoration techniques. These evolving procedures offer hope and options for those facing the dual challenges of cancer treatment and fertility preservation [30,31,32,33].

The use of GnRH agonists as a pharmacological strategy for fertility preservation during chemotherapy, aimed at minimizing the number of follicles that can be damaged by chemotherapy and thereby reducing the risk of ovarian damage, is still the subject of experimental investigation and debate [34,35,36,37]. These agents induce an initial ‘flare-up’ effect characterized by a transient increase in gonadotropins, a phenomenon that lasts for about two weeks and subsequently decreases due to downregulation of receptors [38]. GnRH agonists, including triptorelin, leuprolide, goserelin, and buserelin, are thought to reduce the risk of ovarian failure by creating a prepubertal hormonal environment and decreasing utero-ovarian perfusion [39]. Nevertheless, these agents have not received FDA approval specifically for fertility preservation and are often used “off label”.

**Figure 1:**
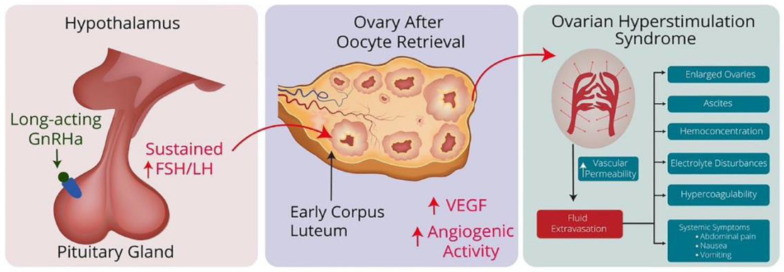
Hypothetical pathway of GnRH agonist-induced OHSS: Long-term administration of GnRHa triggers a flare-up effect by stimulating the pituitary gland, leading to a sustained increase in FSH and LH levels. These increased FSH and LH levels influence the corpus luteum in the ovaries after oocyte retrieval and cause them to release angiogenic factors such as VEGF. This increase in angiogenic factors increases vascular permeability, leading to fluid accumulation in the abdominal cavity and other areas, along with hemoconcentration, hypercoagulability and electrolyte disturbances, the characteristic features of OHSS. Adapted from Ingold et al. [5].

Although still considered experimental, physicians are increasingly using combined strategies for fertility preservation, such as the administration of a long-acting GnRH agonist after COS for fertility preservation by oocyte and/or embryo cryopreservation. However, this approach has been associated with the occurrence of OHSS [5,6]. The mechanism is a sustained increase in gonadotropins after treatment, which stimulates luteinizing hormone (LH) receptors in the ovaries and leads to an overproduction of angiogenic factors, eventually resulting in OHSS (Figure 1).

For cancer patients undergoing fertility preservation, the urgency of starting chemotherapy poses a significant clinical dilemma. The development of OHSS may necessitate delaying critical cancer treatments, which can affect patients’ prognosis [40,41]. While OHSS is usually manageable with intensive medical intervention, its severe manifestations carry significant risks, including thromboembolism, renal failure, and respiratory distress syndrome [42].

## 7. GnRH Antagonists for Ovarian Suppression: A Comprehensive Clinical Evaluation

Gonadotropin-releasing hormone antagonists, which were developed from the natural GnRH decapeptide with various amino acid substitutions, have proven to be a promising alternative in reproductive medicine and gynecology. 

Originally, GnRH antagonists were characterized by complex structures that led to adverse histamine-related side effects such as edematogenic reactions and anaphylaxis [43]. However, advances in pharmaceutical research have led to the development of improved GnRH antagonists that do not have these adverse effects. The newer generation of antagonists, characterized by specific amino acid changes and structural improvements, has an improved safety profile and greater efficacy [44]. 

GnRH antagonists such as cetrorelix, ganirelix, and degarelix have high receptor affinity and inhibitory activity, making them candidates for a range of clinical applications. Cetrorelix and Ganirelix in particular have been shown to be effective in controlling ovarian stimulation protocols, preventing premature LH surges in ART.

The administration of long-acting GnRH agonists initially triggers a ‘flare-up effect’ in hormone levels in the first few days after use. This effect is followed by a suppression of the hormone axis, which usually occurs one to two weeks later. Theoretically, the benefits of ovarian suppression would only be realized after this suppression phase. In clinical practice, treatment protocols vary considerably between medical centers [45]. One notable example is the timing of the first dose of the agonist. In some cases, it is administered at the same time as the start of chemotherapy. This timing means that the patient may be undergoing chemotherapy precisely during the flare-up phase triggered by the GnRH agonist. In contrast to GnRH agonists, GnRH antagonists compete directly with natural GnRH for receptor binding. This competitive inhibition leads to a rapid and sustained reduction in follicle-stimulating hormone (FSH) and LH levels, bypassing the ‘flare-up effect” typically associated with agonist use [38].

The use of GnRH antagonists is also effective in preventing the early onset of OHSS and reducing its severity. This intervention works by inhibiting the release of LH from the pituitary gland, which in turn accelerates luteolysis [46]. This process leads to a significant decrease in serum VEGF [47].

Although the benefits of using GnRH analogs to preserve ovarian function are unproven, GnRH antagonists represent a compelling option for fertility preservation, especially when used in combination with other methods, such as following controlled ovarian stimulation for cryopreservation of oocytes and/or embryos, particularly in scenarios where minimizing the risk of OHSS is of paramount importance. Their rapid suppression of gonadotropin secretion without causing a ‘flare-up’, makes them a preferred alternative to GnRH agonists. This is particularly important in sensitive cases, such as cancer patients undergoing fertility preservation, where minimizing the risk of OHSS is a major concern.

## 8. Conclusions

In conclusion, there have been significant advances in the field of fertility preservation, particularly in relation to patients undergoing treatments such as chemotherapy who are at risk of ovarian failure. The strategic use of various fertility preservation methods, such as cryopreservation of oocytes and embryo cryopreservation and the administration of GnRH agonists, has opened up new possibilities for improving future pregnancy outcomes.

However, these interventions are not without challenges. The use of long-acting GnRH agonists, for example, is still considered experimental and controversial, as their ‘flare-up’ effect raises concerns about OHSS risk. While GnRH antagonists represent a promising approach to reducing the incidence and severity of OHSS, their use requires careful timing and monitoring.

The discussion around these interventions underscores the importance of a tailored, patient-centered approach to fertility preservation. Collaboration between oncologists, reproductive physicians, and patients is essential to developing effective, safe, and patient-specific fertility preservation strategies. The ultimate goal remains to provide patients undergoing potentially gonadotoxic treatment with safe and effective fertility preservation options that ensure a better quality of life after treatment and the ability to meet their reproductive goals in the future.
